# Screening a library of approved drugs reveals that prednisolone synergizes with pitavastatin to induce ovarian cancer cell death

**DOI:** 10.1038/s41598-019-46102-1

**Published:** 2019-07-03

**Authors:** Marwan Ibrahim Abdullah, Mohammed Najim Abed, Farhat Khanim, Alan Richardson

**Affiliations:** 1 Institute for Science and Technology in Medicine, Guy Hilton Research Centre, Keele University, Thornburrow Drive, Stoke-on-Trent, ST4 7QB UK; 2 Al-Salam Teaching Hospital, Nineveh Health Directorate, Ministry of Health, Mosul, Iraq; 3 School of Biosciences, University of Birmingham, Birmingham, B15 2TT UK; 4 University of Mosul, College of Pharmacy, Mosul, Iraq

**Keywords:** Ovarian cancer, Targeted therapies

## Abstract

The survival rate for patients with ovarian cancer has changed little in the past three decades since the introduction of platinum-based chemotherapy and new drugs are needed. Statins are drugs used for the treatment and prevention of cardiovascular diseases. Recent work from our laboratory has shown that pitavastatin has potential as a treatment for ovarian cancer if dietary geranylgeraniol is controlled. However, relatively high doses of statins are required to induce apoptosis in cancer cells, increasing the risk of myopathy, the most common adverse effect associated with statins. This makes it desirable to identify drugs which reduce the dose of pitavastatin necessary to treat cancer. A drug-repositioning strategy was employed to identify suitable candidates. Screening a custom library of 100 off-patent drugs for synergistic activity with pitavastatin identified prednisolone as the most prominent hit. Prednisolone potentiated the activity of pitavastatin in several assays measuring the growth, survival or apoptosis in several ovarian cancer cells lines. Prednisolone, alone or in some cases in combination with pitavastatin, reduced the expression of genes encoding enzymes in the mevalonate pathway, providing a mechanistic explanation for the synergy.

## Introduction

Ovarian cancer is a group of heterogeneous diseases which share the same anatomical location^1^. It is the most lethal gynaecological cancer, causing the deaths of more than 4000 patients annually in the UK^2,3^. Generally, ovarian cancer treatment includes surgery to reduce the tumour mass and chemotherapy, which is most often carboplatin and paclitaxel^4^. Despite considerable improvements in the prognosis of patients with several other solid tumours, ovarian cancer survival rates have changed little in the past three decades since the introduction of platinum-based chemotherapy. The development of resistance to chemotherapy plays a key role in limiting long-term patient survival. The recent introduction of PARP inhibitors offers cause for considerable optimism, although these drugs show less activity in patients with a functional homologous recombination pathway^5^. Therefore, new therapeutic strategies are needed for the treatment of ovarian cancer, and especially for advanced and drug-resistant disease^6^.

Statins are drugs used to treat elevated cholesterol. Their widespread use in this setting has allowed the generation of epidemiological evidence which suggests that their use is also associated with reduced cancer mortality^7^. Statins inhibit hydroxymethylglutarate Coenzyme A reductase (HMGCR), the rate limiting step in the mevalonate pathway. This pathway leads not only to the production of cholesterol, but also isoprenoids which are used to anchor Ras family GTPases to cell membranes. Statins trigger apoptosis in several types of cancer cells^8^. Recently, published data from our laboratory showed that pitavastatin has promising anti-tumour activity against ovarian cancer xenografts^9^. However, prospective clinical trials of statins in cancer have largely been unsuccessful. We have identified three reasons likely to explain this lack of clinical activity (reviewed in^10^). Firstly, the dose of statins used in many trials were comparable to those used to treat hypercholesterolaemia, yet the statin concentration achieved in plasma following such doses falls well below that required to induce apoptosis in cancer cells *in vitro*^11^. Secondly, many of the statins have a relatively short half-life, and once-daily dosing is inadequate to maintain the continual inhibition of HMGCR that we have shown to be necessary to induce apoptosis^12,13^. The choice of statin tested in clinical trials has been, in our opinion, uniformly inappropriate^10^. Hydrophilic statins are less potent in a cancer setting, while lipophilic statins are the most potent anti-cancer agents^12^ but they are generally associated with short metabolic half-lives^13^. Pitavastatin is the only lipophilic statin with an adequate half-life to maintain continual inhibition of HMGCR using a practical dosing schedule. Consequently, we consider that high doses of pitavastatin, administered twice daily, are the most likely to succeed. Lastly, dietary sources of geranylgeraniol can interfere with the anti-tumour activity of pitavastatin, suggesting diet should be controlled during therapy^9^. These observations suggest clinical trials of pitavastatin are warranted.

The use of statins at a high dose and with continuous exposure, as we propose, brings with it an increased risk of myopathy, the most common adverse effect associated with statin use. In some rare cases this can lead to rhabdomyolysis and the incidence of this is likely to increase if high statin doses are used^14^. It is, therefore, desirable to identify compounds which synergize with the anti-tumour activity of pitavastatin in order to reduce the dose needed and potentially reduce the incidence of myopathy. Combination therapies are among the most successful forms of treatment of cancer. Tumours, especially in adults, are associated with multiple mutations and intratumoral clonal heterogeneity is often observed as a result of several different pathological mechanisms participating in their evolution^15^. Thus, drug combinations can be more successful than single agents^16^. Drug combinations can also simultaneously affect different signalling pathways in individual cancer cells, potentially leading to synergistic activity. Drug combinations may also reduce the emergence of drug-resistant subpopulations. Lastly, there is an historical precedent for the use of drug combinations because many chemotherapeutic regimens incorporate several different drugs. Therefore, combining drugs offers the prospect of obtaining a more sustained clinical response. We, and others, have already shown that bisphosphonates such as zoledronatate potentiate the activity of statins^11,17–21^ and dipyridamole has been shown to potentiate the activity of atorvastatin^22^.

To identify additional drugs which might be synergistic with pitavastatin, we screened a library of 100 off-patent clinically approved drugs in combination with pitavastatin using cell growth assays. This library was designed to allow testing of the drugs at clinically achievable concentrations^23^. This library has previously been screened to identify niclosamide, an anti-helminthic drug, as a potential therapy for multiple myeloma as it killed several cell lines at clinically achievable non-toxic concentrations^23^. In this study, we show that prednisolone potentiates the activity of pitavastatin against a panel of ovarian cancer cell lines.

## Material and Methods

### Compounds

Pitavastatin (Livalo, Adooq), Prednisolone, farnesol, geranylgeraniol and mevalonate (Sigma-Aldrich) were prepared as 20 mM stock solution in DMSO. The custom-made drug repurposing library (FMC1) was provided by Dr. Farhat Khanim, School of Biosciences, University of Birmingham and is comprised of off-patent, mainly orally bioavailable drugs, at a concentration which is a multiple of each drug’s plasma C_max_ observed in patients^23^.

### Cell culture

A panel of ovarian cancer lines (Cov-318, Cov-362, Ovcar-3, Ovcar-4, Ovsaho) were incubated in a humidified incubator at 37 °C in 5% CO_2_ atmosphere. Cell lines were maintained in RMPI-1640 (Ovcar-3, Ovcar-4, Ovsaho) or DMEM (Cov-318 and Cov-362) supplemented with 10% fetal bovine serum, 2 mM L-Glutamine and 50 IU/ml penicillin/streptomycin. Medium for Ovcar-3 cells was additionally, supplemented with 0.01 mg/ml bovine insulin and 1 mM sodium pyruvate.

### Screening the drug library with pitavastatin

Ovcar-4 cells were seeded (5000 cells/well) in a 96-well plate. The next day, cells were exposed to vehicle (DMSO), pitavastatin (10 µM), a library compound, or a combination of pitavastatin and a compound from the library. The experimenters were blind to the identity of the drugs which were each given an anonymized code. Each drug was tested in triplicate in two independent experiments. After 72-hours incubation, cells were fixed and relative surviving cells were estimated by staining with SRB and measuring A_570_ as described previously^24^. The Bliss independence criterion was used to estimate the expected effect of the drug combination if the drugs interacted additively^25^. The “Bliss excess” was calculated from the difference between the observed effect and expected additive effect.

### Cell growth assays

Human ovarian cancer cells (5000 cells/well) were plated in 96-well plates. The following day, cells were exposed 18 different concentrations of the drugs for 72-hour, with the exception of Cov-318 and Cov-362 cells which were incubated for 120-hour because of their slower growth rate. Cells were stained with sulforhodamine B as previously described^24^. IC_50_ values and Hill coefficients were determined using Graphpad Prism 6.

To evaluate synergy, complete concentration-response curves for pitavastatin were measured in the absence or presence of a fixed concentration of prednisolone. Combination indices were calculated as described^26^ at fraction affected = 0.5. Some cells were also exposed to 20 μM mevalonate, 10 μM farnesol (FOH), or 10 μM geranylgeraniol (GGOH) as indicated.

### Spheroid cultures

GravityTRAP ULA Plates (InSphero) were pre-wet with 40 µl of medium before seeding 500 cells in 70 µl growth medium per well. Following centrifugation (ALC PK120 Centrifuge, 1 min at 900 rpm), the plates were returned to the incubator. After 3–5 days, spheroids were observed. Thereafter, drugs were added in 30 µL of growth medium. Ovcar-4 or Cov-362 cells were incubated for 72 or 120 hours, respectively before relative ATP levels were measured by addition of 25 µL of cell Titer-Glo Luminescent assay reagent (Promega, Madison, WI, USA). The effect of the combination was compared to that expected for an additive interaction using the Bliss independence criterion as described above.

### Caspase-Glo3/7 assays

For each experiment two 96 well plates containing 5000 cells per well in 80 µl of growth medium were prepared. After 48 hours, caspase activity was measured in one plate by addition of 20 µL of Caspase-Glo 3/7 reagent (Promega, Madison, WI, USA). The second plate was stained with SRB as described above and the caspase activity normalised to the SRB staining.

### Annexin V/propidium iodide staining

Ovcar-4 or Cov-362 cells were seeded at density of 2 × 10^6^ cells per well of a 6 well plate in 2 mL of growth medium. The following day, drugs were added in 20 μL of growth medium to the indicated final concentration. Ovcar-4 and Cov-362 cells were incubated with drugs for 48 and 72 hours, respectively. The cells were labelled using an annexin-V FITC kit (Miltenyi biotech). Cells were trypsinized, washed in ice-cold PBS and centrifuged at 300 × *g* for 5 minutes. The pellets were re-suspended in 1 ml of binding buffer, and centrifuged for 10 minutes at 300 × *g*. The pellets were re-suspended in 100 µl of annexin V binding buffer and 10 µl of annexin V fluorochrome were added to each sample and incubated for 10 minutes in dark at room temperature. The washing step were repeated with 1 ml of annexin V binding buffer. Lastly, the cells were re-suspended in 500 µl annexin V Binding Buffer and 5 µl of propidium iodide (1 µg/ml) added before analysis by flow cytometry. The viability of cells was defined as alive (annexin V-negative and PI-negative), early apoptotic cells (annexin V-positive and PI-negative), late apoptotic cells or dead cells (annexin V-positive and PI-positive) and necrotic cells (annexin V-negative and PI-positive).

### Western blotting

Ovcar-4 or Cov-362 cells were seeded at density of 2 × 10^5^ cells per well of a 6 well plate in 2 mL of growth medium. 20 μL of medium containing pitavastatin or prednisolone or a combination of both were added to the indicated final concentration. After 48-hour (Ovcar-4) and 96-hour (Cov-362) incubation with drugs, floating and adherent cells were collected. Cell lysates were prepared as described^27^ and protein concentration measured by BCA assay. Equal masses of the sample proteins were separated by SDS-PAGE and transferred to a PVDF membrane. The membrane was incubated overnight at 4 °C with primary antibody: anti-PARP (1:1000) (Cell Signaling Technology); anti-HMGCR (1/1000) (Abcam); anti-GGTI-β subunit (1/1000) (Santa Cruz); anti-RABGGTII-β subunit (1/1000) (Santacruz); anti-MVD (1/1000) (Abcam), anti-IDI1 (1/2000) (Abcam) anti-HMGCS (1/1000) (Abcam) or with anti-GAPDH antibody (1:5000) (Millipore) as loading control. Proteins were visualised using peroxidase-conjugated secondary antibodies and Uptilight™ Ultra WB Chemiluminescent Substrate (Interchim, France).

### Statistical analysis

Student’s paired t-test with Welch corrections or one-way ANOVA followed by Tukey’s post hoc performed for multiple statistical comparisons were performed as indicated. Differences considered statistically significant at *P* < 0.05.

## Results

### Testing a library of compounds in combination with pitavastatin

Ovcar-4 cells, which are considered representative of high grade serous ovarian cancer^28^, were used to test the effect in cell growth assays of pitavastatin alone and in combination with individual compounds from a library (FMC1) of off-patent, licensed drugs^23^. Six compound showed significant growth inhibitory activity against Ovcar-4 cells when they were tested as single agents at a concentration similar to their C_max_ achieved in patients. Five compounds potentiated the effect of pitavastatin, namely prednisolone (71.6 µM, Bliss excess = 0.29), rifampicin (12.2 µM, Bliss excess = 0.19), praziquantel (3.5 µM, Bliss excess = 0.16), flutamide (6.22 µM, Bliss excess = 0.23) and mefenamic acid (41.4 µM, Bliss excess = 0.21). Prednisolone showed the most significant synergistic effect (70 μM, Bliss excess ~0.4) and was selected for further analysis.

### Single agent activity in panel of ovarian cancer cell line

The single agent activity of prednisolone was determined using a panel of ovarian cancer cells considered representative of high grade serous ovarian cancer, namely Ovcar-4, Ovcar-3, Ovsaho, Cov-318 and Cov-362 cells^28^. Prednisolone, as a single agent, showed weak growth inhibitory activity in all ovarian cancer cell lines at concentrations up to 500 μM and an accurate IC_50_ could not be determined using pharmaceutically-relevant concentrations. This agrees with other studies that report that corticosteroids have insignificant growth inhibitory activity against solid tumours^29^. In contrast, and as we have previously reported^9,17^, pitavastatin inhibited the growth of tested cell lines with an IC_50_s ranging from 1.1 to 4.8 µM (Table 1).

**Table 1:** Single agent potency of pitavastatin in cell growth assays.

Cell lines	Pitavastatin IC_50_ (µM)	Number of experiments
Cell line	IC_50_ (μM)	n
Cov-318	3.1 ± 0.6	4
Cov-362	3.3 ± 0.7	4
Ovcar-3	4.1 ± 0.1	3
Ovcar-4	4.8 ± 0.6	3
Ovsaho	1.1 ± 0.3	4

The potency of pitavastatin was measured in cell growth assays. The IC_50_ is reported as the mean (±S.D.) of the indicated number (n) of experiments.

### Pitavastatin combination with prednisolone

**Figure 1:**
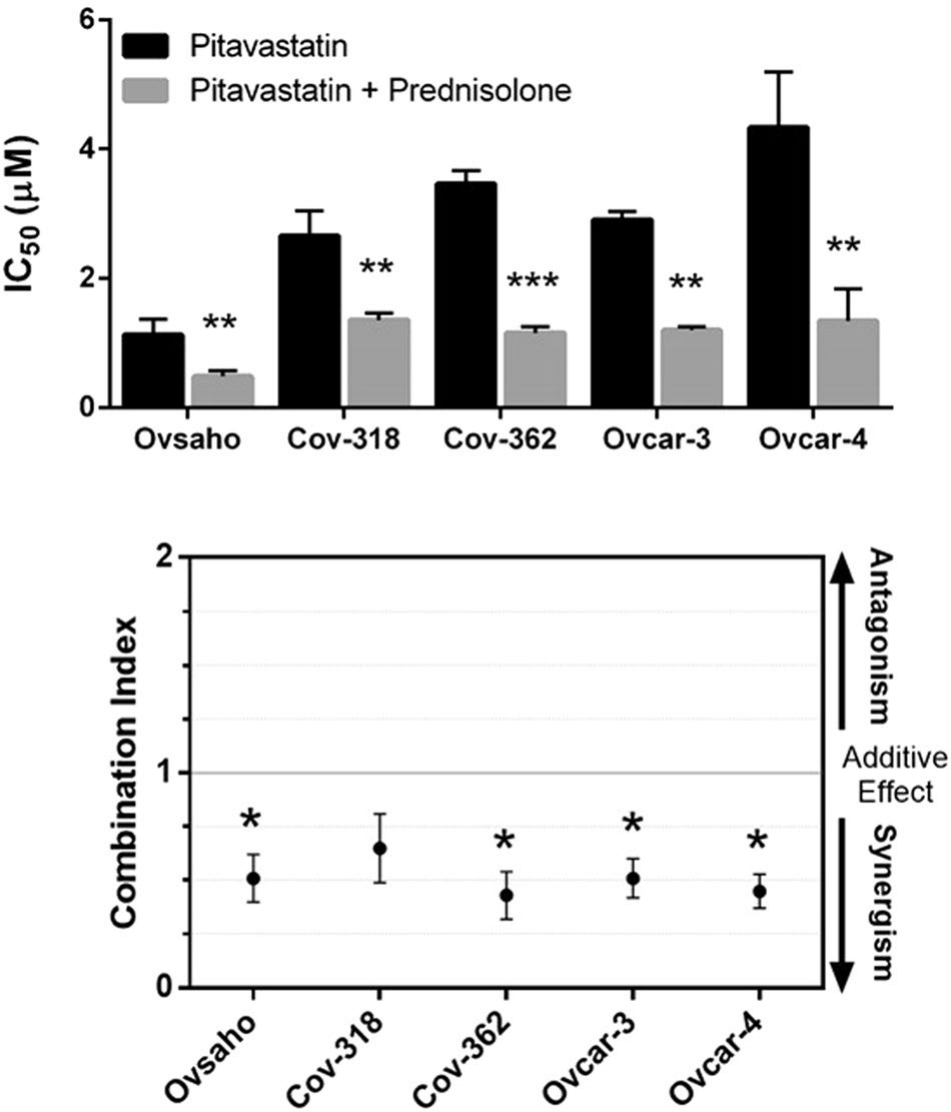
Synergy between pitavastatin and prednisolone in cell growth assays. (**A**) The potency of pitavastatin (IC_50_ in cell growth assays) in the presence and absence of a fixed concentration of prednisolone (70 µM) against a panel of ovarian cancer cell lines. The IC_50_ was significantly increased by inclusion of prednisolone in all the tested cell lines (**, *** paired t-test, *P* < 0.01, 0.001, respectively, n = 3). (**B**) Combination indices (CI) (Mean ± SD, n = 3–4) were calculated for the above data and are quoted at a fraction affected of 0.5. *, ** differed significantly from unity where indicated (*^,^**P ≤ 0.05, 0.01, respectively).

To confirm the results of the screen, a range of concentrations of pitavastatin were combined with a fixed concentration of prednisolone (70 µM) and their activity assessed in cell growth assays. At this concentration, prednisolone has no measureable effect as a single agent, so any change in the apparent potency of pitavastatin must reflect a drug interaction. Prednisolone potentiated the activity of pitavastatin against all the ovarian cancer cell lines that were tested (Ovsaho, Cov-318, Cov-362, Ovcar-3 and Ovcar-4), with significant reduction in pitavastatin IC_50_s (Fig. 1A). To confirm this formally, combination indices were calculated. Significant synergy between prednisolone and pitavastatin was observed in all the cell lines (Fig. 1B).

### Effect of mevalonate pathway intermediate metabolites on the combination

**Figure 2:**
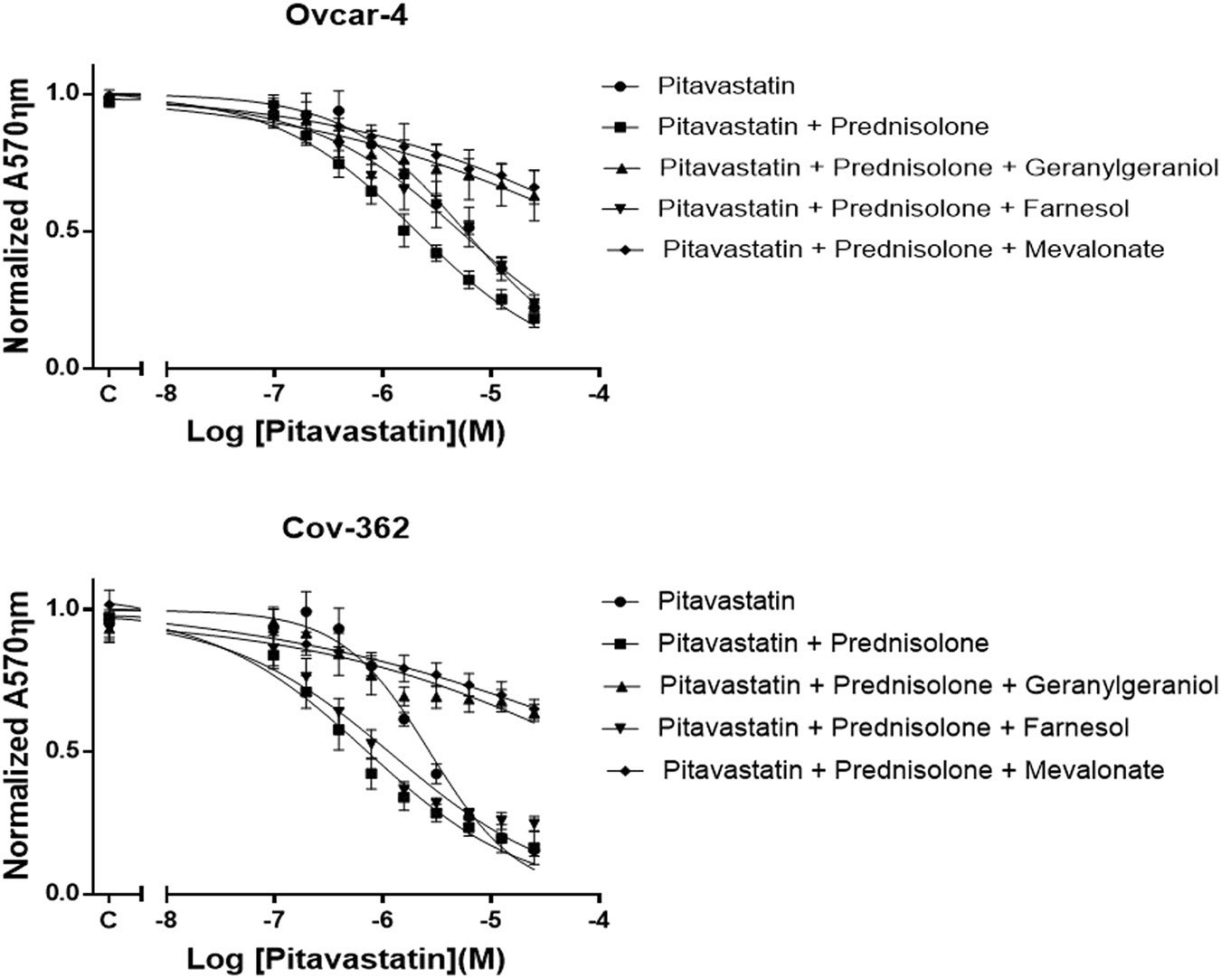
Mevalonate pathway intermediates reduce the activity of pitavastatin. The addition of geranylgeraniol (10 µM) and mevalonate (20 µM), but not farnesol (10 µM), suppressed the effect of pitavastatin or the pitavastatin and prednisolone combination. Ovcar-4 and Cov-362 cell lines were exposed to serial dilution of pitavastatin in combination with prednisolone (70 µM) for 72 and 120 hours, respectively. The data is presented as a fraction of the top of the curve calculated by curve fitting (mean ± S.D., n = 3). “C” represents the control cells exposed to solvent alone.

We^9^, and others^30–32^, have previously shown that some mevalonate pathway metabolites downstream of HMGCR including mevalonate and geranylgeraniol reduce the cytotoxic effect of statins. To determine if the anti-proliferative activity of the pitavastatin and prednisolone combination resulted from inhibition of mevalonate pathway, Ovcar-4 and Cov-362 cells were exposed to the drug combination and further supplemented with mevalonate, farnesol or geranylgeraniol. The addition of mevalonate to cells significantly reduced the growth inhibitory activity of drug combination. Furthermore, supplementing the combination with geranylgeraniol but not farnesol also significantly prevent growth inhibition (Fig. 2). These results suggested that activity of combination is mediated mainly through inhibition of mevalonate pathway and most likely through inhibition of geranylgeranylation.

### ATP assay in spheroid cultures

**Figure 3:**
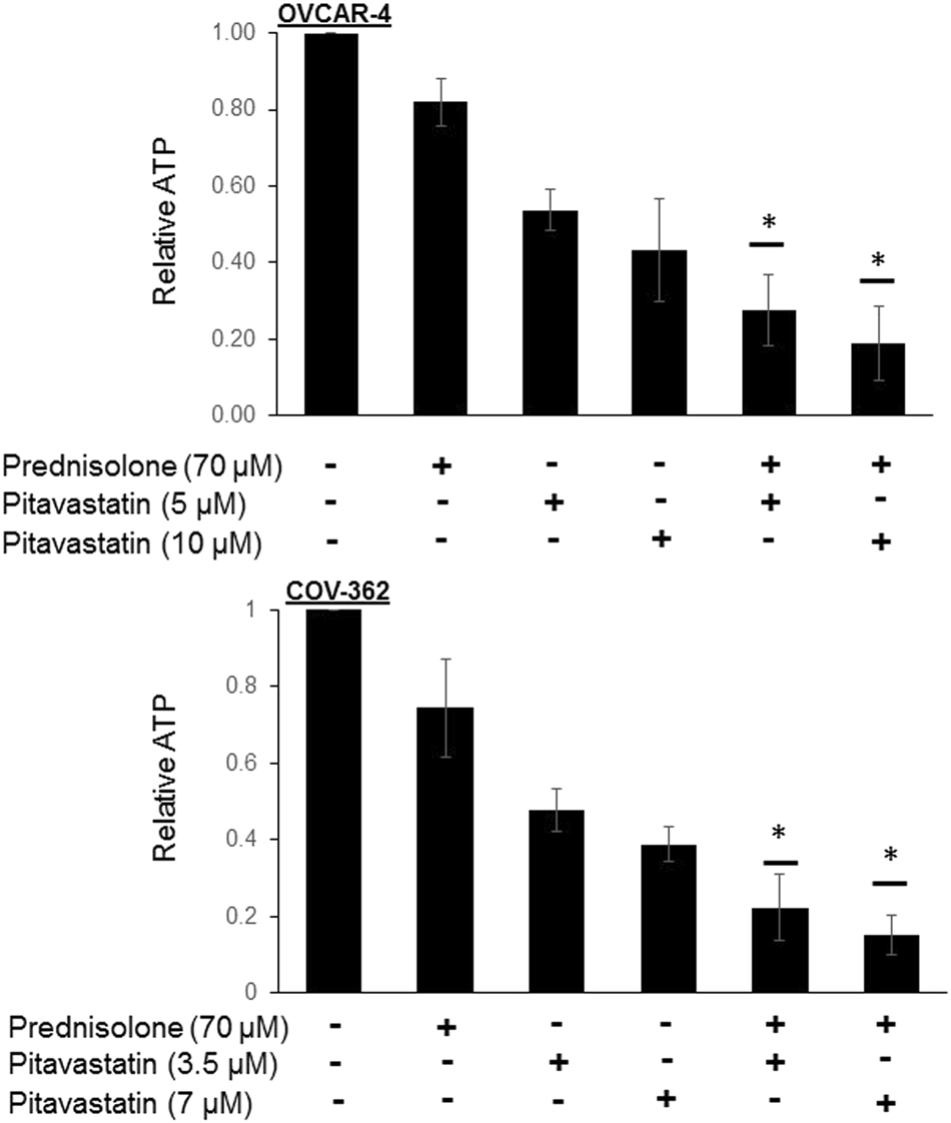
The effect of pitavastatin and prednisolone on spheroids. Spheroids of Ovcar-4 and Cov-362 cells were treated with the indicated drug concentration for 72 hours (Ovcar-4) or 120 hours (Cov-362). The relative viabilities were then measured using CellTiter-Glo assay to measure relative ATP and expressed as fraction of that measured in control samples treated with solvent (mean ± SD; n = 3). The observed effect of the drug combination were compared to the effect expected if the drugs had additive effects which was calculated using the Bliss independence criterion (shown with a line for each drug combination) from the measured effect of the individual drugs in each individual experiment. The results were significantly different from the Bliss expected effect where shown (*P < 0.05; paired t-test).

To recapitulate the 3D architecture of tumours more closely *in vitro*, Ovcar-4 and Cov-362 spheroids were prepared and the effect of the pitavastatin and prednisolone combination was evaluated. ATP was measured as a surrogate of surviving cell number. The combination of prednisolone and pitavastatin reduced ATP significantly more than would have been anticipated if the drugs acted additively (estimated from the Bliss independence criterion; Fig. 3), thereby confirming a synergistic interaction between the two drugs.

### Prednisolone and pitavastatin synergistically induce apoptosis

**Figure 4:**
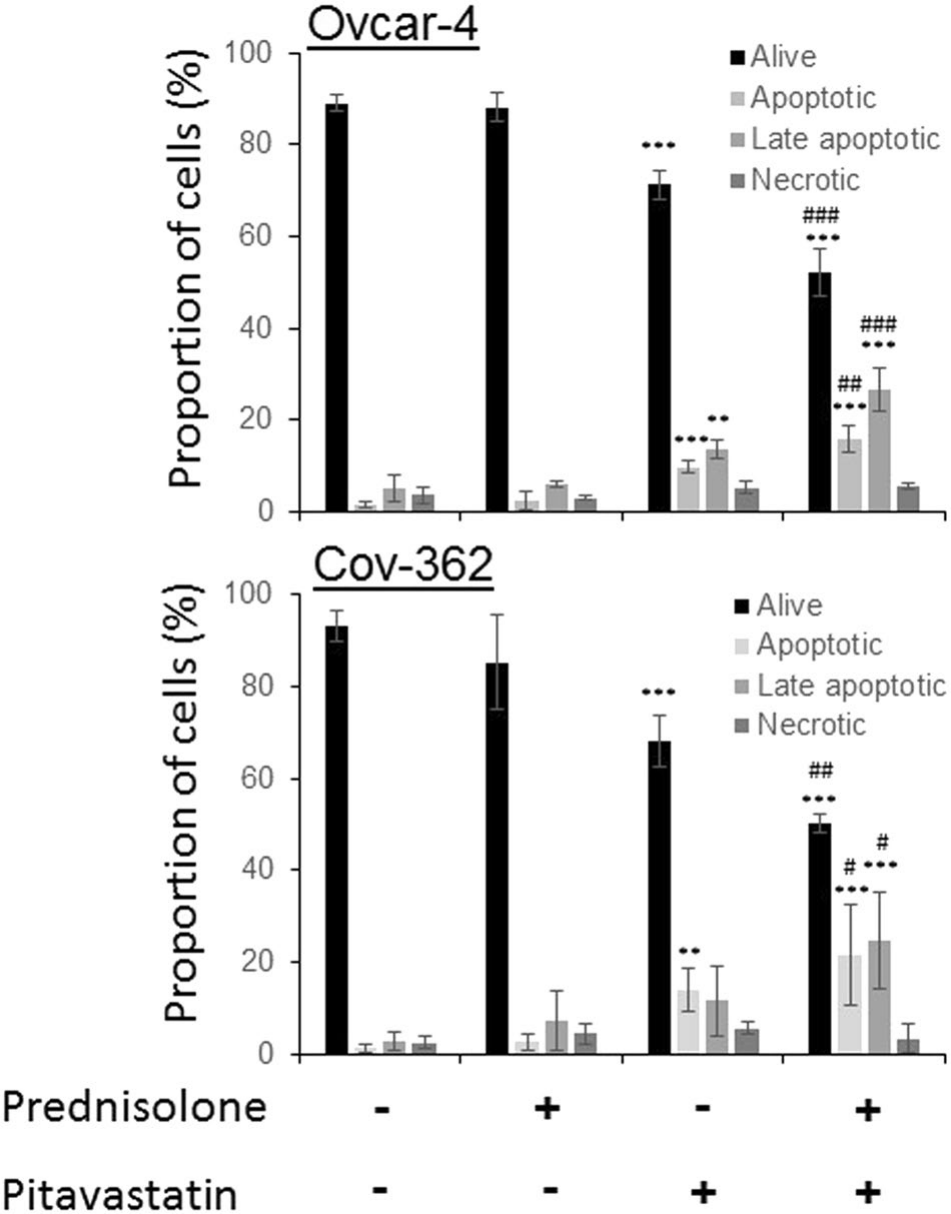
Prednisolone and pitavastatin synergisticially increase staining with annexin V and propidium iodide. Ovcar-4 (**A**) or Cov-362 (**C**) cells were exposed to the indicated drug concentrations for 48 hours, the cells were labelled with annexin V and propidium iodide and assessed by flow cytometry. The results shown are representative of 3 experiments. (**B**,**D**) The percentage of cells in each quadrant were compared with the control untreated cells (*) or with pitavastatin alone (#). The results (mean ± S.D., n = 3) were significantly different were indicated (^*,#^*P* < 0.05; **,^# #^*P* < 0.01; ***,^**###**^, ### < 0.001) (ANOVA test followed by Tukey’s post hoc test).

**Figure 5:**
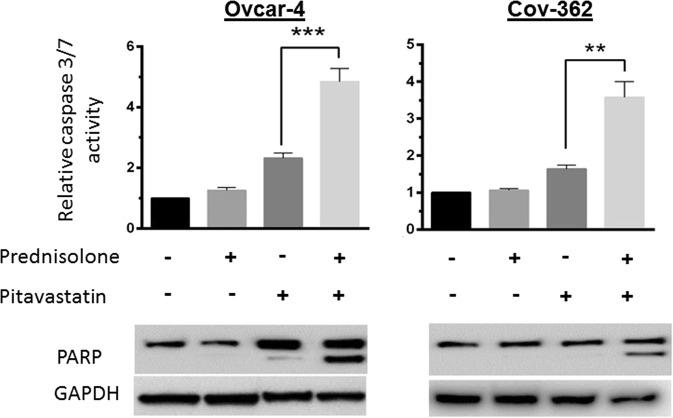
Prednisolone and pitavastatin synergistically increase Caspase 3/7 activity and PARP cleavage. Caspase3/7 activity was measured by Caspase 3/7-Glo assay and the results expressed as fold of control (mean ± SD; N = 3). Cells were treated with of pitavastatin (10 μM) and/or prednisolone (10 μM Ovcar-4, 7 μM Cov-362) for 48 hours (Ovcar-4) or 72 hours (Cov-362). (**P < 0.01, *** < 0.001; t-test, n = 3). PARP was measured by immunoblotting (n = 3).

When Ovcar-4 cells were treated with solvent or with prednisolone and viewed by phase contrast microscopy they retained their original morphology. In contrast, cells exposed to pitavastatin alone detached from the plate surface (Supplementary Fig. 1) were round, shrunken and with blebs and this was more pronounced in cells treated with the drug combination. To determine if these morphological changes resulted from apoptosis, Ovcar-4 and Cov-362 cell lines were exposed to pitavastatin, prednisolone or the combination of the two agents before annexin V and propidium iodide staining was measured by flow cytometry. There were significantly more early apoptotic or late apoptotic dead cells in samples treated with the drug combination than in cells treated with pitavastatin alone (Fig. 4) suggesting that the drug combination synergistically induced apoptosis. To confirm this, caspase-3/7 activity and PARP cleavage were assessed. Although prednisolone had negligible effect on caspase 3/7 activity on its own, the combination of pitavastatin with prednisolone caused significantly more caspase activation than that caused by pitavastatin alone. Consistent with this, immunoblot analysis demonstrated that the prednisolone and pitavastatin combination caused significant accumulation of cleaved PARP that was greater than that observed with each single agent (Fig. 5).

### Identification of a potential mechanism underlying synergy between pitavastatin and prednisolone

**Figure 6:**
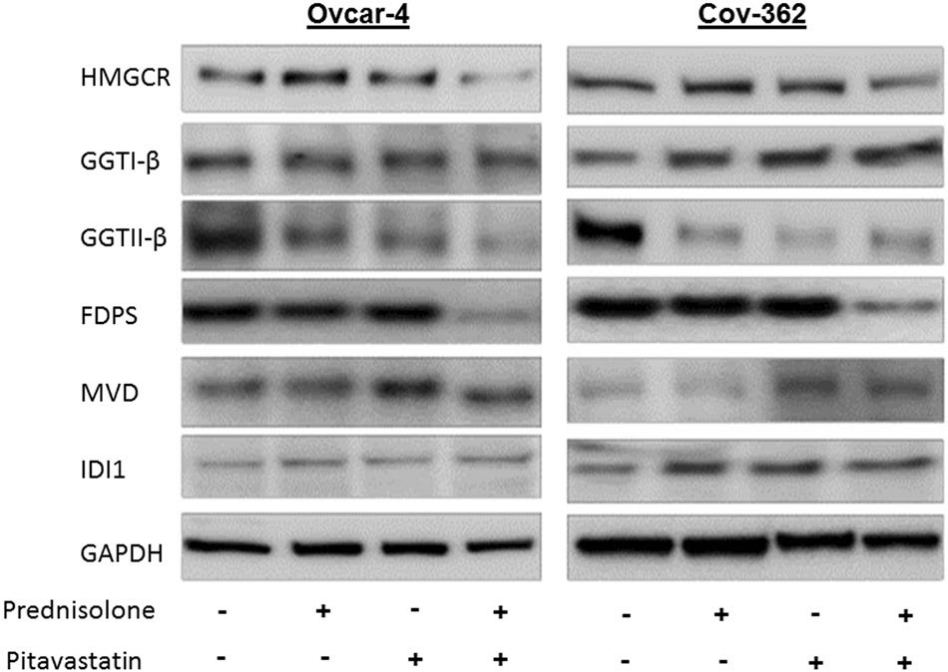
Effect of prednisolone and pitavastatin on mevalonate pathway enzymes. Ovcar-4 and Cov-362 cell line were exposed for 48 hours or 72 hours, respectively, to prednisolone (70 μM) and or pitavastatin (10 μM). The level of mevalonate enzymes was detected by immunoblotting for HMGCR, GGTI-β, GGTII-β, FDPS, MVD, IDI1 and GAPDH, (n = 3).

Prednisolone regulates the expression of genes by binding to the glucocorticoid receptor, a ligand-dependant transcription factor. This raised the possibility that the synergy between pitavastatin and prednisolone occurred as a result of prednisolone-induced changes in gene expression. Previous work^33^ has identified genes whose expression is altered in 3T3-L cells exposed to prednisolone, including some which form part of the mevalonate pathway. The expression of the genes encoding HMGCR, geranylgeranyl transferase I and II (GGTI, GGTII), isopentenyl diphosphate isomerase (IDI1), mevalonate decarboxylase (MVD) and farnesyl diphosphate synthase (FDPS) were reported to be decreased in cells exposed to prednisolone. Consequently, the effect of prednisolone, alone and in combination with pitavastatin, on these genes products was assessed in ovarian cancer cells. Neither pitavastatin nor prednisolone when used as single agents notably altered the levels of HMGCR, FDPS, IDI1, MVD, GGTI-β. However, GGTII-β was reduced upon exposure to either pitavastatin or prednisolone as single agents as well as in cells exposed to the combination of these two drugs. The combination of pitavastatin and prednisolone, but not the single agents alone, caused significant reduction in levels of HMGCR and FDPS (Fig. 6).

## Discussion

Statins in general are well tolerated when used at recommended doses as anti-hypercholesterolemia agent in clinic. However, to cause apoptosis in cancer cells, high doses are likely to be required, which increases the risk of myopathy, creating a challenge for redeployment of statins as chemotherapeutic agent. One strategy to potentially minimize the adverse effects is to identify drugs which synergize with the anti-cancer activity of statins, thereby reducing the dose of statin required. Our screen of approved drugs to discover those which potentiate the activity of pitavastatin identified prednisolone. The synergy between pitavastatin and prednisolone was confirmed in several assays using a panel of ovarian cancer cell and led to the decreased expression of mevalonate pathway genes, providing a potential mechanistic explanation for the synergy.

The synergy between the pitavastatin and prednisolone combination identified in the screen was verified in several assays. This included cell growth assays in monolayers and in 3D cell culture. The increase in apparent potency of pitavastatin in the presence of prednisolone, at a concentration at which itself has minimal effect, provides unequivocal evidence of synergy between the two drugs. Cell death was mediated, at least in part through, induction of apoptosis. A synergistic increase in apoptosis was observed in three separate apoptosis assays (Annexin V labelling, caspase 3/7 activity and PARP cleavage). These observations provides robust evidence that pitavastatin and prednisolone can interact synergistically.

We have previously shown that pitavastatin causes cell death through inhibition of the mevalonate pathway even when pitavastatin is used at relatively high concentration^9,17^. Inhibition of the mevalonate pathway causes disruption of several GTPases which are involved in cell signalling, regulating cell cycle progression and cell survival^34^. Consequently, statins induce apoptosis including an increase in release of mitochondrial cytochrome C to cytosol, and activation of caspases 3, 8 and 9^35,36^. In this study we also found that that the effects of the combination of prednisolone and pitavastatin was also dependant on inhibition of the mevalonate pathway, because geranylgeraniol and mevalonate both reduced the activity of the drug combination. Reminiscent of the effects of statins alone^12,30–32^, farnesol failed to block the activity of the combination against the cancer cells. This suggests that the effects of the drug combination are primarily mediated through inhibition HMGCR and the consequent inhibition of geranylgeranylation. This is also consistent with our previous observation that inhibition of both GGTI and GGTII potentiates the activity of pitavastatin as a single agent^17^.

The foregoing discussion, particularly the effects of geranylgeraniol, strongly argues that the effect of the combination of pitavastatin and prednisolone depends upon its effects on the mevalonate pathway. We have also previously shown that that dual inhibition of the mevalonate pathway, using bisphosphonates to inhibit FDPS, is also synergistic with pitavastatin^17^. To explore the mechanism by which prednisolone was synergistic with pitavastatin in more detail, levels of mevalonate pathway enzymes were investigated by immunoblotting. An earlier study found that prednisolone altered the expression of genes encoding several mevalonate pathway enzymes including HMGCR, GGTI, GGTII, IDI1, MVD and FDPS^33^. Inhibiting the mevalonate pathway by two separate mechanisms provides a potential rationale to explain the synergy we observed between pitavastatin and prednisolone. We found that the prednisolone-pitavastatin combination cause significant reduction in level of HMGCR and FDPS enzymes. It was striking that the combination, but not the single agents, affected the level of these enzymes, consistent with this contributing to the synergy observed between the drugs. A reduction in GGTII-β was observed following exposure to either of the drugs as a single agents, as well as in the combination. Taken together, this strongly suggests that synergy between prednisolone and pitavastatin may result from inhibiting multiple points on the mevalonate pathway. The effect of related steroids on mevalonate pathway enzymes has also been reported by others. Investigation of the short term effects of dexamethasone in rat hepatocytes revealed a reduction in cholesterol synthesis^37^. Dexamethasone also causes down regulation of HMGCR and FTase enzymes activity in rat AR 4-2J cells^38^. Specifically, the authors found that there is significant reduction in FT-α subunit upon treatment of the cells with dexamethasone for 48 hours. In contrast, the β-subunit of the enzyme was either unchanged or slightly reduced. However, it was claimed that even a 50% reduction of FT activity is not sufficient to prevent Ras isoprenylation and Ras protein were even found to accumulate during dexamethasone treatment. Therefore, it is plausible that a relatively small amounts of an active prenyl transferase is sufficient to maintain prenylation process and support cell survival. We have also previously shown that inhibition of geranylgeranyl transferase I and II simultaneously is necessary to synergize with pitavastatin^17^, suggesting that one prenyltransferase may compensate for the reduced activity of one of the other enzymes. These observations suggest that for efficacy in cancer, robust inhibition of the mevalonate pathway is required and may explain why relatively high concentrations of pitavastatin are required to induce apoptosis. In turn, this may also further explain why combinatorial inhibition of the mevalonate pathway potentiates cell death. Although we have shown the combination induces apoptosis, it is also plausible that the combination affects the autophagy pathway which we^12^ and others^39^ have already shown to be affected by statins.

The most straightforward explanation for the changes in the abundance of mevalonate pathway enzymes in cells exposed to prednisolone is that their expression is controlled by the glucocorticoid receptor, a transcription factor to which prednisolone binds. However, the mevalonate pathway is subject to a complex set of regulatory mechanisms which may provide an alternative explanation for the activity of prednisolone. Mevalonate pathway enzymes, particularly HMGCR, are regulated by sterol and non-sterol products of the pathway^40^. The HMGCR enzyme itself is regulated at several levels including regulation of its catalytic activity, its rate of degradation and its rate of synthesis^41^. In particular, sterols and oxysterols, which are product of the mevalonate pathway, play a role in feedback regulation of the mevalonate pathway. Sterols and oxysterols inhibit transcription of HMGCR and other mevalonate pathway genes. They bind to the regulatory proteins SCAP and Insig and prevent the translocation of SREBP to the Golgi complex where it otherwise undergoes activation by proteolytic cleavage to allow transcription of mevalonate pathway genes^42,43^. Oxysterols can also directly affects the activity of HMGCR, squalene monooxygenase, FDPS and several enzymes in cholesterol biosynthetic pathway^44^. Oxysterols also accelerate the degradation of HMGCR through sterol-sensing domain in a fashion that depends on the mevalonate pathway regulator Insig^40^. Therefore, it is reasonable to speculate that prednisolone, which also has a sterol ring structure, may mimic sterols and oxysterols and binds to some of these sterol regulatory binding sites. In this manner, prednisolone may reduce the levels of HMGCR and FDPS enzymes either by decreasing transcription or increasing the degradation of the enzymes, or a combination of both mechanisms. In this scenario, adding prednisolone to pitavastatin may prevent reactivation of the mevalonate pathway which would otherwise occur as a result of reduced cholesterol synthesis following inhibition of HMGCR by pitavastatin. In other words, maintenance of feedback inhibition by prednisolone provides one explanation for the reduction in mevalonate pathway enzymes observed in this study. Further work will be necessary to uncover the detailed basis of the regulation of the mevalonate pathway by prednisolone.

Our previous observations^9,10,12,17^ suggest that pitavastatin warrants clinical evaluation in ovarian cancer. The current work suggests that it may be appropriate to evaluate the combination of prednisolone and pitavastatin in clinical trials. The concentration of prednisolone we have used in these studies, although relatively high, is comparable to those clinically achievable (C_max_) using a relatively high dose of prednisolone^45^. We do not consider, however, that prednisolone warrants exploration as a single agent in ovarian cancer. Although steroids can induce apoptosis in lymphoid cells^46^ steroids as monotherapy show only limited activity in breast and prostate cancers but not in other cancer types^47,48^. In agreement, we found a very limited effect of prednisolone as a single agent on ovarian cancer cell lines. A further possibility is to consider more complex combinations. We have previously shown synergy between pitavastatin and bisphosphonates^17^ and other workers have also reported activity of bisphosphonates as single agents against ovarian cancer xenografts in mice^39^. Thus, it may be worthwhile considering the clinical use of a combination of pitavastatin with both a bisphosphonate and with prednisolone. All these drugs are approved for clinical use so there is no regulatory barrier preventing this in principle. It is also worth considering whether statins should be combined with chemotherapy. Although some workers have reported mild synergy between lovastatin and either carboplatin or paclitaxel^39^, we previously mostly observed additivity^12^. Furthermore, we observed profound antagonism if cells were exposed to simvastatin prior to carboplatin^12^, possibly reflecting cell cycle arrest reducing the activity of the chemotherapeutic agents. Thus, we currently do not favour combining pitavastatin with a chemotherapeutic agent.

In conclusion, drug repositioning provides a great opportunity to find new indications for existing drugs. The anti-cancer activity of pitavastatin is potentiated significantly by prednisolone by augmenting inhibition of the mevalonate pathway. Clinical trials of prednisolone with pitavastatin in patients with ovarian cancer may be warranted.

## Supplementary information

Supplementary info
