# Supplementary material for: Screening a library of approved drugs reveals that prednisolone synergizes with pitavastatin to induce ovarian cancer cell death
Source: Sci Rep. 2019 Jul 3;9:9632. doi: 10.1038/s41598-019-46102-1 (PMC6610640; doi:10.1038/s41598-019-46102-1)

**Screening a library of approved drugs reveals that prednisolone synergizes with pitavastatin to induce ovarian cancer cell death.**

Marwan Ibrahim Abdullah, Mohammed Najim Abed, Farhat Khanim^,^ Alan Richardson

**Supplementary Information**

Supplementary Figure 1.


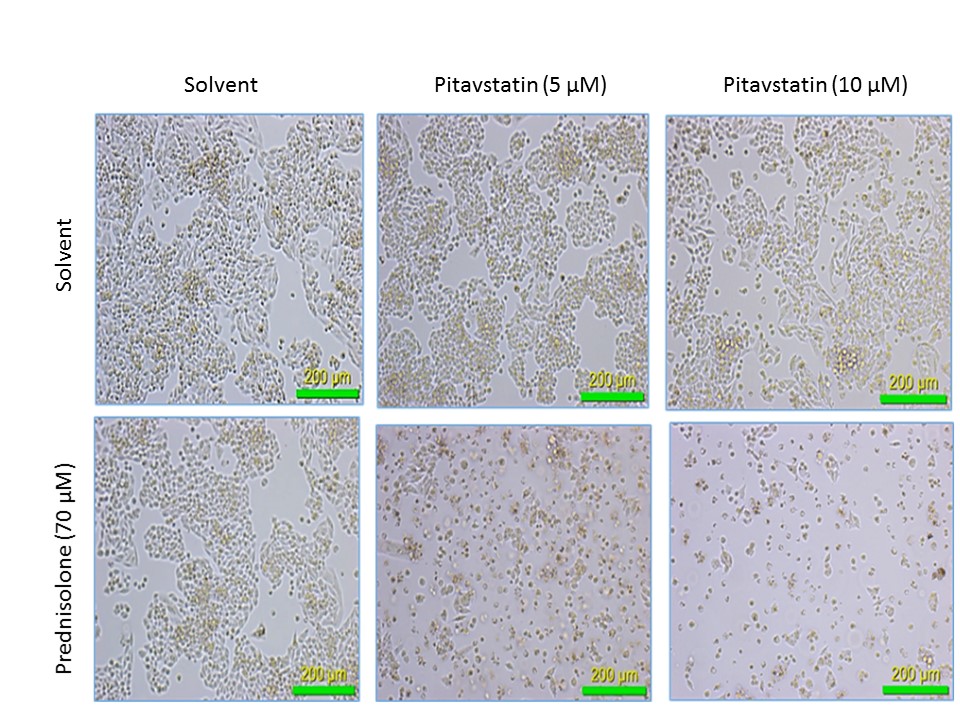

Supplement: Supplementary file 1 — Supplementary info [file 41598_2019_46102_MOESM1_ESM.docx]
